# 2-Mercaptobenzimidazole clubbed hydrazone for Alzheimer’s therapy: *In vitro*, kinetic, *in silico*, and *in vivo* potentials

**DOI:** 10.3389/fphar.2022.946134

**Published:** 2022-08-09

**Authors:** Farida Begum, Najeeb Ur Rehman, Ajmal Khan, Sajid Iqbal, Rehan Zafar Paracha, Jalal Uddin, Ahmed Al-Harrasi, Muhammad Arif Lodhi

**Affiliations:** ^1^ Department of Biochemistry, Abdul Wali Khan University Mardan, Mardan, Khyber Pakhtunkhwa, Pakistan; ^2^ Natural and Medical Sciences Research Centre, University of Nizwa, Nizwa, Birkat-ul-Mouz, Oman; ^3^ Department of Industrial Biotechnology, Atta-ur-Raman School of Applied Biosciences (ASAB), National University of Sciences and Technology (NUST), Islamabad, Pakistan; ^4^ School of Interdisciplinary Engineering and Sciences (SINES), National University of Sciences and Technology (NUST), Islamabad, Pakistan; ^5^ Department of Pharmaceutical Chemistry, College of Pharmacy, King Khalid University, Abha, Saudi Arabia

**Keywords:** acetylcholinesterase, Alzheimer’s disease, 2-mercaptobenzimidazole derivatives, molecular docking, molecular dynamic (MD) simulation

## Abstract

Alzheimer’s is a type of dementia that affects the affected person’s thinking, memory, and behavior. It is a multifactorial disease, developed by the breakdown of the neurotransmitter acetylcholine via acetylcholinesterase (AChE). The present study was designed to evaluate potential inhibitors of acetylcholinesterase that could be used as a therapeutic agent against Alzheimer’s disease (AD). For this course, synthetic compounds of the Schiff bases class of 2-mercaptobenzimidazole hydrazone derivatives (**9–14**) were determined to be potent acetylcholinesterase inhibitors with IC_50_ values varying between 37.64 ± 0.2 and 74.76 ± 0.3 μM. The kinetic studies showed that these are non-competitive inhibitors of AChE. Molecular docking studies revealed that all compounds accommodate well in the active site and are stabilized by hydrophobic interactions and hydrogen bonding. Molecular dynamics (MD) simulations of selected potent inhibitors confirm their stability in the active site of the enzyme. Moreover, all compounds showed antispasmodic and Ca^2+^ antagonistic activities. Among the selected compounds of 2-mercaptobenzimidazole hydrazone derivatives, compound **11** exhibited the highest activity on spontaneous and K^+^-induced contractions, followed by compound **13**. Therefore, the Ca^2+^ antagonistic, AChE inhibition potential, and safety profile of these compounds in the human neutrophil viability assay make them potential drug candidates against AD in the future.

## 1 Introduction

Alzheimer’s is a neurodegenerative disease characterized by memory loss, behavioral disturbances, and cognitive problems (Deture and Dickson, 2019; Kaur et al., 2021). Alzheimer’s disease (AD) is related to insufficiency of functions in the basal forebrain and cortex (Shekari and Fahnestock, 2019; Liu et al., 2022). Cholinergic neurotransmission impairment negatively affects learning and memory loss in AD patients. It has been reported that inhibition of these cholinesterases, which leads to the activation of cholinergic function, may be an effective method for AD treatment (Marucci et al., 2021; Rong et al., 2021). Cholinesterases like acetylcholinesterase (AChE) are the key enzymes for regulating neurotransmission by hydrolysing acetylcholine (ACh) in cholinergic neurons (Massoulié et al., 1993; Aramjoo et al., 2021). AChE is a membrane-bound multi-subunit enzyme mainly present in cholinergic neurons, the brain and muscles. In the human brain, the majority of the AChE is found in the membrane-bounded tetrameric G4 form, and its level decreases as the degeneration of the neurons occurs (Manna et al., 2008). It plays a significant role in the management of various physiological reactions by hydrolysing ACh in cholinergic synapses (Schetinger et al., 2000; Kaur et al., 2021). Usually, in the brains of AD patients, the activity of AChE remains unchanged or declines (Kumar et al., 2018; Karthika et al., 2022). Currently available synthetic acetylcholinesterase inhibitors, including galanthamine, rivastigmine, donepezil, and tacrine, have been clinically used for AD treatment (Moreira et al., 2022). However, these drugs have therapeutic activity along with side effects such as short duration of biological action, gastrointestinal disturbance, low bioavailability, and hepatotoxicity (Reza et al., 2018). Due to the adverse effects of previously approved drugs, new AChE inhibitors are of great interest for the treatment of AD (Kabir et al., 2021). Schiff bases are significant organic compounds involved in several biological activities like α-glucosidase, urease, β-glucuronidase, and antiglycation (Khan et al., 2011; Taha et al., 2019a; Taha et al., 2019b).

The study of new biologically important Schiff bases has been drawing the attention of pharmacists and chemists (Matela, 2020). Previous studies indicate that the lone pair of nitrogen atoms of the azomethine group of Schiff bases is chemically and biologically significant (Ashraf et al., 2011). The nitrogen atom also participates in the creation of hydrogen bonds with active cell constituent centers and interferes with cell functions. The azomethine or imine (–C=N–) group in Schiff bases is also found to be a versatile pharmacophore for the development of new drugs (Hameed et al., 2017; Vanitha et al., 2022). Gallic hydrazide-derived Schiff bases are ketone derivatives with antioxidant activity that also show AChE inhibition and are considered as a potential treatment for AD. Schiff bases are also involved in biological activities like antimicrobial (Da Silva et al., 2011), anticancer (Tadele and Tsega, 2019) and herbicidal activities (Zhu et al., 2016). Moreover, it has been investigated that Schiff base compounds have antiviral (Kumar et al., 2010), anthelmintic (Husain et al., 2018), antiprotozoal (Kordestani et al., 2021) and anticonvulsant activities (Nilkanth et al., 2020).

The current study was designed to evaluate AChE inhibitory activity and the kinetics of compounds (**9–14**). Moreover, binding interactions and stability of these potent inhibitors were determined using molecular docking and molecular dynamic (MD) simulation.

## 2 Materials and methods

During this experimental study, analytical grade solvents were used. The purity of products was monitored through alumina plates, and the melting point was determined using a hot-stage Gallenkamp melting point apparatus (Loughborough, United Kingdom). Generally, n-hexane and ethyl acetate solvent medium were used to check reaction through plates. The progress of the reaction was monitored by thin-layer chromatography. An ultraviolet lamp was used as a visualizing agent.

The whole reaction was carried out in clean glassware with specific catalysts in basic or acidic conditions. All synthesized compounds were characterized by using different spectroscopic techniques such as 13C NMR 1H NMR replace with 13C NMR 1H NMR were performed on the Advance Bruker AM at 300, 400, and 500 MHz. Thin Layer Chromatography (TLC) was performed on pre-coated silica gel aluminum plates with dimensions of 3 × 8 cm (Kieselgel 60, 254, E. Merck, Germany). The chromatogram was visualized with dual wavelengths of UV at 254 and 365 nm. The melting point was found on the Gallon kemp apparatus.

### 2.1 General procedure for the synthesis of novel hydrazone derivatives on 2-mercaptobenzimidazole (9–14)

2-Mercaptobenzimidazole based hydrazone derivatives synthesis were carried out through multistep reactions. First, 2-mercaptobenzimidazole was refluxed with bromoethane in basic conditions (KOH) in ethanol with equimolar amounts for about 10 h. After completion of the reaction, the reaction mixture was filtered. The filtrate so obtained was kept until the whole ethanol was evaporated and got shiny white needle-like crystals of 2-ethylthio benzimidazole. In the second step, 2-ethylthio benzimidazole was taken in a round bottom flask and refluxed with ethyl chloroacetate (dropwise) using anhydrous potassium carbonate in DMF (solvent) for about 15 h. After completion of the reaction, the product (2-(2-(ethylthio)benzimidazolyl) acetate) was obtained and got through a separating funnel in semisolid form. In the third step, 2-(2-(ethylthio)benzimidazolyl) acetate was refluxed in methanol with hydrazine hydrate for about 10 h. The product, 2-((ethylthio)benzimidazolyl) acetohyrazide get was poured into ice-cold water until a precipitate was formed. The precipitate was filtered and then dried in an open atmosphere. In a fourth step, (ethylthio)benzimidazolyl) acetohyrazide was dissolved in methanol with 2-3 drops of acetic acid (catalyst) on a hotplate. After 10 min, aldehyde was added and refluxed into the whole mixture for about 5–6 h. The progress of the reaction was monitored by TLC. After completion of the reaction, the mixture was poured into ice-cold water until a precipitate was formed. The precipitate was collected by filtration, washed with water, and then dried in an open atmosphere.

### 2.2 General

All chemicals used in the current study were of analytical grade. The acetylcholine iodide (Cat. No. A7000-25G), acetylcholinesterase EC 3.1.1.7 (Cat. No. C3389-2KU), DTNB (5-dithiobis 2-nitrobenzoic acid) (Cat. No. D8130-10G), Galanthamine (Cat. No. 69353-21-5) (MO, United States). Di-Sodium hydrogen phosphate (Cat. No. 558-79-4), sodium phosphate monobasic dehydrate buffer (Cat. No. 1342-35-0) and ethanol (Cat. No. 64-17-5) were purchased from Sigma Aldrich. All compounds of 2-mercaptobenzimidazole hydrazone derivatives (**9–14**) were synthesized as described earlier (Yousaf et al., 2020).

### 2.3 *In vitro* acetylcholinesterase inhibition assay

AChE inhibitory activity was evaluated as described previously with few modifications using a microtiter plate reader (Molecular Device, CA, United States) (Hasan et al., 2005). Initially, 100 mM of sodium phosphate buffer (140 µl) pH 8, 0.25 mM DTNB (10 µl), 0.5 mM of synthetic compounds dissolved in 20 µl of ethanol and AChE (20 µl) were mixed and incubated for 15–20 min at room temperature in a microtiter plate. Finally, 10 µl of substrate ACh (0.4 mM) was added and incubated at room temperature for 4–6 min. The reaction started after the addition of ACh was hydrolyzed by AChE in the presence of DTNB; a yellow-colored 5-thio-2-nitrobenzoate anion was formed, which indicates the reaction completion and was read at a wavelength of 412 nm. All experiments were performed in triplicate and were analyzed using the SoftMax Pro6.3 program (Molecular Device, CA, United States).

Percentage inhibition was calculated by

% inhibition = (O.D control-O.D test well) / O.D control)×100.

### 2.4 Evaluation of kinetics parameters

The IC_50_ is the quantity of a test compound required for 50% inhibition. IC_50_ was determined at various concentrations, and EZ-Fit EK was employed to calculate the IC_50_ values of the test compounds (Perrella Scientific Inc., Amherst, MA, United States).

Enzyme-substrate (ES) is the complex of AChE and acetylcholine, while P represents the product formed after the reaction’s completion. Dissociation constant values were calculated through Dixon plots, their secondary replots, and the Lineweaver-Burk plot (Khan et al., 2021).

The values of *K*_m_, *K*_i_, and *V*_max_ were determined from Dixon and Lineweaver-Burk plots using no linear regression equation. *K*_i_ values were determined by using the Lineweaver-Burk plot; first, values of 1*/*V_max*app*_ were found at every intersection point of the lines of each test compound concentration on the y-axis. On the Lineweaver-Burk plot, the slope of each line obtained as a result of the compound was plotted against different concentrations of the test compounds.

### 2.5 Statistical analysis

GraFit software (Khan et al., 2021) was utilized for plotting graphs. The values of the correlation coefficients, slopes, intercepts, and their standard errors were determined by the linear regression equation using the same program.

### 2.6 Homology modelling

In the current study, electric eel AChE was used in the laboratory to perform an AChE inhibitory assay, but for computational analysis, we used hAChE (4EY6). Therefore, homology modeling was performed to check the similarity between the electric eel and hAChE.

**FIGURE 1:**
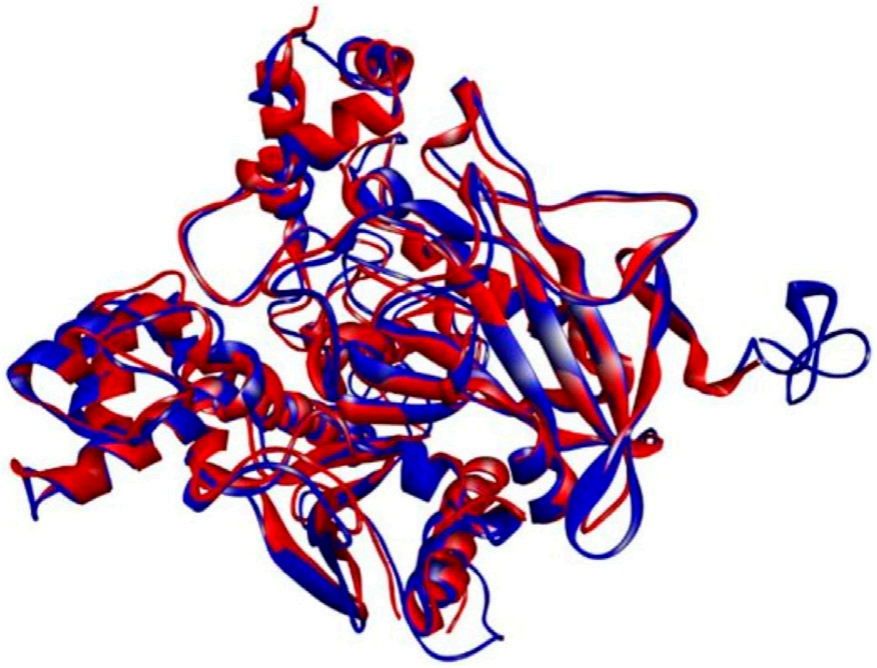
Structural alignment of electric eel AChE (red) and hAChE (blue).

The eel 3D crystal structure is not available. So, the 3D structure of electrophorus electric eel AChE was predicted using the Iterative Threading Assembly Refinement (I-TASSER) (https://zhanglab.ccmb.med.umich.edu/I-TASSER/). I-TASSER prioritized the five best models from which the model with the best C-Score of −0.15 was selected with an accuracy value of 0.69 and a root mean square deviation (RMSD) value of 8.2 Å. C-score is a confidence score for assessing the quality of prioritized protein structure models by I-TASSER. The 3D model of electrophorus electric eel was then superposed on the 4EY6 retrieved from PDB (Figure 1) to check the similarity between both structures. Both the model and 4EY6 structures were exactly aligned with each other. Therefore, (4EY6) was selected for further molecular docking and MD simulation study.

### 2.7 Molecular docking

Molecular docking was conducted to predict the binding interactions between AChE and tested compounds using the MOE-Dock (Molecular Operating Environment-docking) (www.chemcomp.com) program. The results of docking studies were correlated with the experimental outcomes. The ligands and receptors were prepared before being subjected to molecular docking.

### 2.8 Preparation of protein

The 3D structure of 4EY6 at a resolution of 2.40 Å was retrieved from the protein databank (PDB) (https://www.rcsb.org/) and was used in further analysis. Before docking, the AChE was prepared by playing the molecule to add hydrogen atoms, correct bonds, and complete chains (Martínez-Rosell et al., 2017). All crystallographic molecules such as water were removed from AChE, then energy was minimized after the 3D protonation using the default parameters of the MOE energy minimization algorithm.

### 2.9 Preparation of ligands

The 3D structures of all tested compounds were generated using a molecular building program in MOE. A database was designed and the energy of the tested compounds was minimized up to a 0.05 gradient by employing the MMFF94x force field. All the AChE interacting compounds were docked into the AChE binding pocket as described earlier (Fang et al., 2014). Molecular docking protocols were validated by re-docking a co-crystallized ligand (galanthamine) in the active pocket of AChE 4EY6 (Supplementary Figure S1).

### 2.10 MD simulation of the inhibitor with AChE

Top docking score poses of compound **11** and **13** complexes with AChE were subjected to MD simulations using the GROMACS 5.1.2 software package (Berendsen et al., 1995). The protein-ligand complex system has been prepared by using the pdb2gmx module in the GROMACS package. The GROMACS OPLS-AA/L force field (Berendsen et al., 1995) was utilized for parameterization and the generation of the topology of the native protein. Ligand topologies were separately prepared using the swissparam web server (Zoete et al., 2011). Each complex was solvated in a cubic box with a size of (10 ⨯10 ⨯10), encompassing approximately 33436 TiP3P water molecules. To neutralize the whole system with a salt ion environment, the Genion module in GROMACS was utilized under physiological conditions (NaCl 0.15 M). Finally, nine sodium ions were added to neutralize the ligand enzyme complex. The steepest descent minimization algorithm with PR (position restrain) of ligands and protein was used to minimize the complex until the maximal force of 10 kJ/mol (Nath et al., 2017) by eradicating errors in atomic position and structural disputes such as bond angle, bond length, and also the structural clashes between the ions, the position of water molecules, and the protein complex (Yang et al., 2019). Two additional equilibration steps were performed in a sequential process: 1) at constant temperature (300 K) by utilizing isothermal-isochoric ensemble (NVT) [No. of Particles (N) system volume (V) and temperature (T)] programs and 2) under fixed stabilized 1 bar pressure through NPT [No. of particles (N), system Pressure (P), temperature (T)] ensemble system. The ensemble would run 50,000 steps for 0.1 ns. After equilibration steps, the temperature and pressure stabilized complex system was subjected to position restraining where the molecules of solvent in the cubic box were fully dissolved with the protein-ligand complex system. Finally, the position restrained protein-ligand complex was used to simulate for 10,000,000 ps at 300 K (V-rescale thermostat) temperature, atmospheric NPT ensemble pressure (Parrinello-Rahman barostat), and periodic boundary conditions for 0.002 ps using leap-frog algorithms. In order to constrain all the hydrogen bonds, the LINC algorithm was applied during the whole equilibration (Yuan et al., 2012), whereas the Particle Mesh Ewald (PME) module with 0.16 Å Fourier grid spacing has been functionalized (Petersen, 1995). The entire trajectories have been saved at a frequency of 2 fs time step rate during the simulation for further analysis.

### 2.11 Cytotoxicity testing

A cytotoxicity test was performed to determine the adverse effect of tested compounds on humans using neutrophil cells.

#### 2.11.1 Viability of human neutrophil cells

Heparinized whole blood of healthy volunteers was obtained from a local blood bank, and neutrophils were separated using the Choudhary et al. protocol with slight modifications as previously described (Choudhary et al., 2005).

#### 2.11.2 Assay procedure

The isolated neutrophils (1×10^7^ cells/ml) were incubated for 30 min with inhibitors; after that, 0.25 mM WST-1 (water-soluble tetrazolium salt) was added, the microtiter plate was incubated at 37°C in a water bath shaker for 3 h and absorbance was calculated at 450 nm using a microplate reader (Spectra-MAX, CA, United States). The absorbance is the mean of five experimental replicates.

The % viability of neutrophils was measured by the following formula:

Percentage viability: {(O.D test×100/O.D control)-100}-100.

### 2.12 Calcium ion channel blocking and spasmolytic activities

The spasmolytic activity of the inhibitors was evaluated using isolated contracting rabbit jejunum (Rahman et al., 2019). Rabbits used in the current study were provided by Aga Khan Medical University, Karachi, Pakistan, weighing between 1.5 and 2.0 kg. The standard operating protocol has already been defined (Choudhary et al., 2005).

The rabbit jejunum displayed spontaneous rhythmic contractions under these experimental conditions, allowing us to observe spasmolytic activity directly without the use of an agonist. Calcium antagonistic activity was also performed and confirmed the relaxation of 80 mM K^+^ produced contraction.

## 3 Results and discussion

The chemistry of these compounds is in the Supplementary Material.

### 3.1 Biology

The synthetic 2-mercaptobenzimidazole hydrazone derivatives were screened against AChE. All compounds (**9–14**) showed significant inhibitory activities with IC_50_ values between 37.64 ± 0.2 µM to 74.76 ± 0.3 µM in different concentrations (Table 1). The most potent compound **11** of the series, with an IC_50_ value of 37.64 ± 0.2 µM, showed excellent inhibitory activity against AChE as compared to other compounds of the series but showed less potency as compared to standard galanthamine (IC_50_ = 26.03 ± 0.4 µM). The inhibitory potential of this compound may be due to the presence of strong electron-donating –OCH_3_ groups at positions-2 and 4 in the benzene ring. The methoxy group has more electron-donating capacity at the ortho and para positions as compared to other positions. Compounds **13** and **12** are the second most active compounds in the series with IC_50_ values of 50.58 ± 0.3 and 51.07 ± 0.5 µM exhibit good inhibitory activity. This may be due to the presence of electron-donating groups, two –OH groups in compound **13** and two –OCH_3_ in compound **12**. As previously reported, that –OH and OCH_3_ are strong electron-donating groups and are involved in biological activities (Ullas et al., 2020). Compounds **10** and **14** are the third and fourth most potent compounds in the series, with the same IC_50_ (51.23 ± 0.2 µM) values. The activity of compound **10** could be associated with the electron-donating –Cl group, and the potency of compound **14** might be due to the delocalization of π-electrons in the anthracene ring, which is highly conjugated and show–M effect (Yousaf et al., 2020).

**TABLE 1:** Inhibitory activities of 2-mercaptobenzimidazole hydrazone derivatives (**9–14**) against AChE.

Compound	R	Chemical formula	IC_50_ ± SEM (µM)	Docking score
**9**	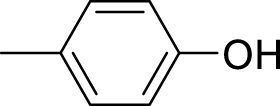	C_18_H_18_N_4_O_2_S	74.76 ± 0.3	−9.816
**10**	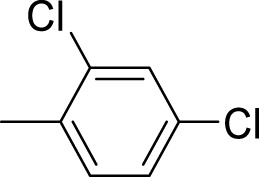	C_18_H_16_Cl_2_N_4_OS	51.23 ± 0.2	−12.950
**11**	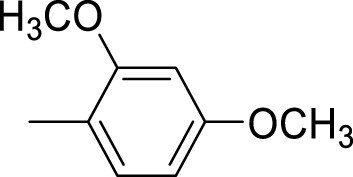	C_20_H_22_N_4_O_3_S	37.64 ± 0.2	−15.455
**12**	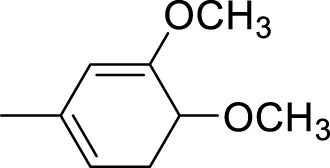	C_20_H_22_N_4_O_3_S	51.07 ± 0.5	−11.019
**13**	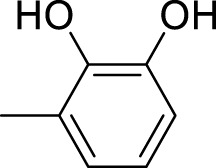	C_18_H_18_N_4_O_3_S	50.58 ± 0.3	−14.560
**14**	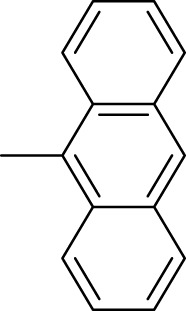	C_26_H_22_N_4_OS	51.23 ± 0.2	−12.572
Galantamine	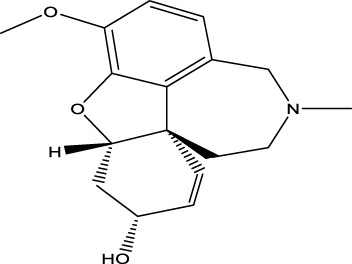		26.03 ± 0.4	−17.301

The compound **9,** with an IC_50_ value of 74.76 ± 0.3 µM contains an –OH group at the *ortho* position in the benzene ring, which is mostly associated with enhanced enzymatic activity, while in some cases, decreasing enzyme activity has also been observed. The increased activity of –OH group may be related to the involvement of oxygen and hydrogen interactions with various residues of the enzyme. In conclusion, the enhanced activities of these compounds may be due to the presence of electron-donating groups like –OCH_3_, –OH, and –Cl.

### 3.2 Kinetic study

During kinetic studies, the reaction rate was measured and the effect of different concentrations of a substrate on the enzyme was investigated. The kinetic study of enzymes helped to determine the catalytic mechanism of enzymes. The substrate concentrations and enzyme activity relationship was first suggested by Leonor Michaelis and Maud Menten (Johnson and Goody, 2011). The rate of reaction [V] was plotted against various substrate concentrations [S] at constant enzyme concentration. Initially, the rate of reaction [V] increases as substrate concentration increases, and at higher [S], the enzyme becomes saturated, and the rate of reaction reaches *V*_max*,*_ which is referred to as the maximum rate of reaction. These synthetic compounds inhibited the enzyme AChE in a dose-dependent manner with the values of *K*_*i*_ (14.01–24.36 µM) (Table 2).

**TABLE 2:** Inhibition and kinetic parameters data of AChE in the presence of compounds (**9–14**).

Compound	*K*_i_ (µM) ± SEM	*K*_*m*_ (mM)	*K*_*m*_ *app* (mM)	*V*_max_ (µmol/min)^−1^	*V*_*maxapp*_ (µmol/min)^−1^	Type of inhibition
**9**	24.36 ± 1.2	0.12	0.12	5.0	2.0	Non-competitive
**10**	17.01 ± 1.3	0.12	0.12	5.0	2.1	Non-competitive
**11**	14.01 ± 1.6	0.12	0.12	5.0	2.6	Non-competitive
**12**	19.22 ± 0.9	0.12	0.12	5.0	2.6	Non-competitive
**13**	19.03 ± 1.7	0.12	0.12	5.0	2.3	Non-competitive
**14**	19.23 ± 1.9	0.12	0.12	5.0	2.3	Non-competitive
Galanthamine	10.03 ± 0.7	0.12	0.12	5.1	2.6	Non-competitive

**FIGURE 2:**
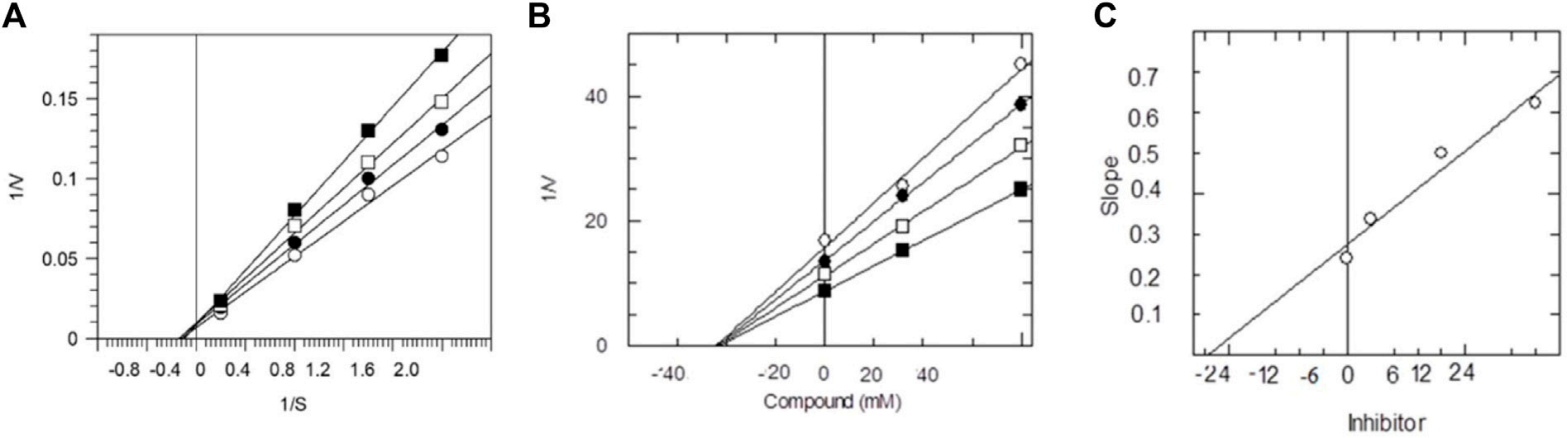
The inhibition of AChE by compound **9**, **(A)** is the reciprocal plot (Linewear-Burk) between initial velocities and four fixed ACh conc. in the absence (■) and presence of 100 µm (□), 150 µM (●), 175 µM (○) of compound **9**. **(B)** Dixon plot of reciprocal between initial velocities and different concentrations of compound **9** at constant ACh concentrations, (■) 100 µM*,* (□) 150 µM, (●) 175 µM and (○) 200 µM. **(C)** Plot between different concentrations of compound **9** and slope.

The dissociation constant *K*_*i*_ values were found using three different methods. Primarily, from the Lineweaver-Burk plot, which is the reciprocal rate of reaction (1/V) versus reciprocal of substrate concentration (1/S), helps to determine the effect of inhibitor on the values of *K*_*m*_ and *V*_max_. Secondly, the Dixon plot is the plot between the reciprocal of initial velocities (1/V) versus different concentrations of the compound. Finally, from secondary replot, the slopes of each line versus various concentrations of all compounds. Kinetic studies were carried out to determine the inhibition type, which helps to specify the mechanism of action of enzyme inhibition and the inhibitor binding moieties. All three applied method results indicated non-competitive types of inhibition against AChE by synthetic compounds **9–14**. In all cases, *K*_*m*_ remains constant while *V*_max_ decreases. The values of *K*_*i*_ *, K*_*m*_ *, V*_max_, and *V*_*maxapp*_, along with the type of inhibition, are shown in Table 2. The graphical representation of steady-state inhibition for 2-mercaptobenzimidazole hydrazone derivatives (**9–14**) against AChE enzyme is shown in Figure 2.

### 3.3 Molecular docking results

Molecular docking was performed to find the binding interactions of the synthetic compounds in the active site of the enzyme AChE. All compounds were well accommodated in the binding pocket of 4EY6. Herein, the selected compounds based on docking score and IC_50_ values were considered.

**FIGURE 3:**
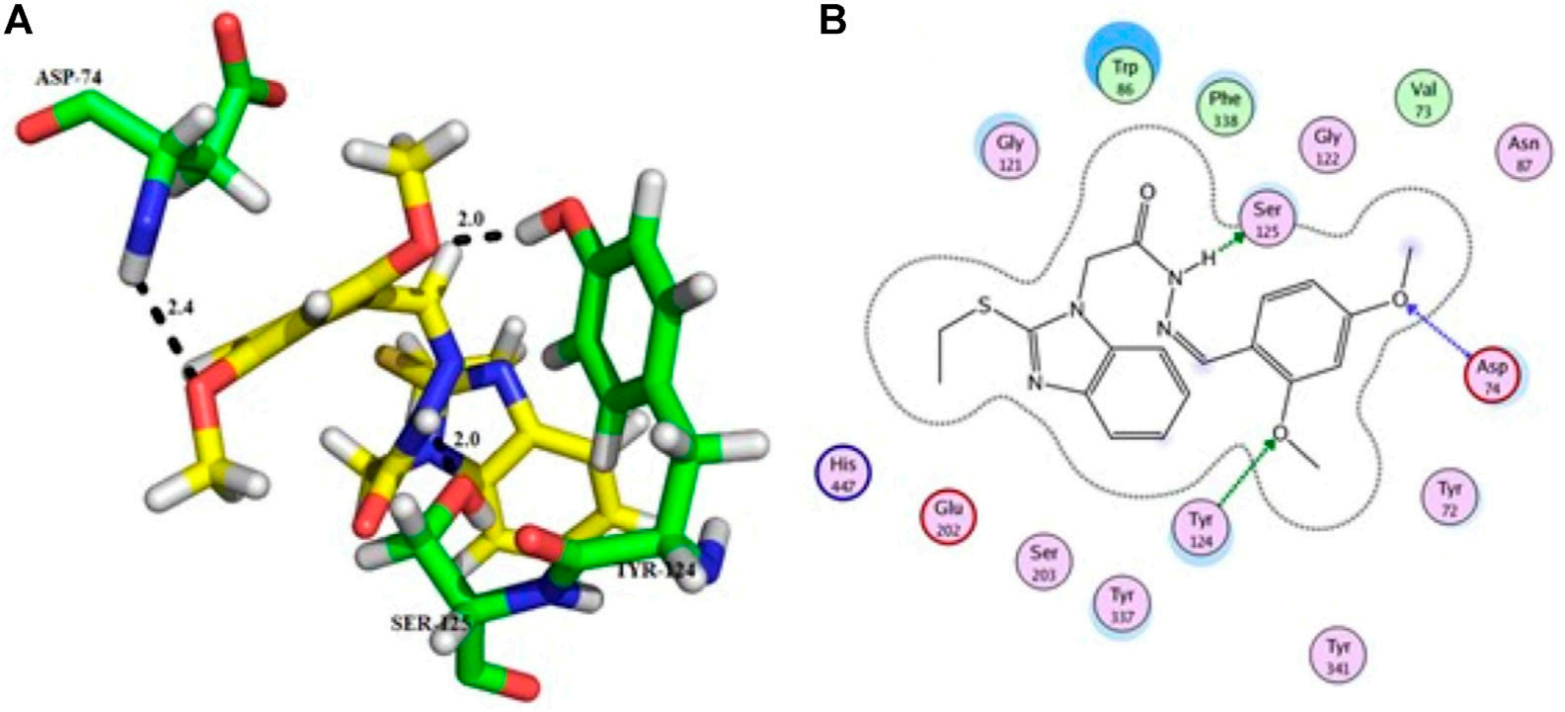
Showing the molecular docking conformation of compound **11 (A,B)** in the active site of AChE.

**FIGURE 4:**
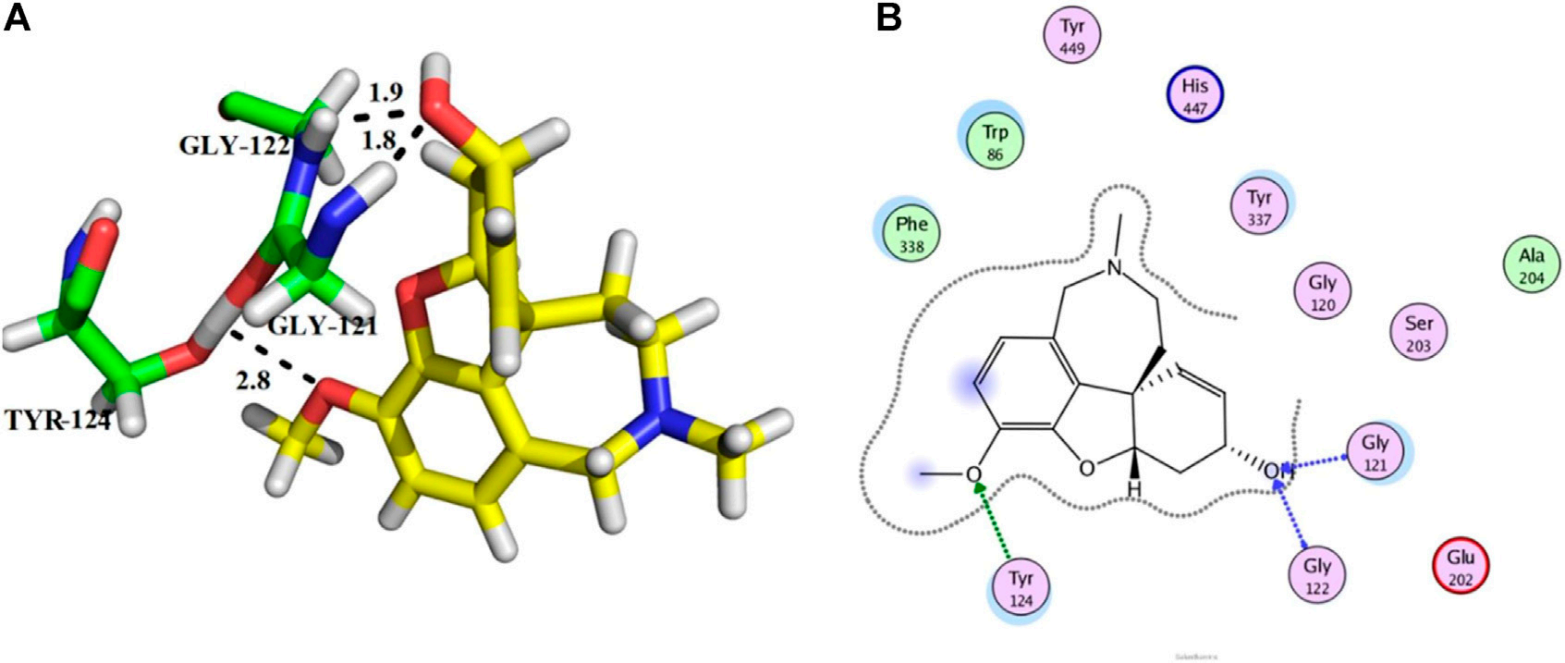
Showing the docking conformation of galanthamine (**A,B)** (positive control) in the active site of AChE.

Compound **11** was the most potent compound of the series (IC_50_ = 37.64 ± 0.2 µM, docking score = −15.455), which formed three hydrogen bonds with Ser-125, Tyr-124, and Asp-74. The amido group of compound **11** formed a strong hydrogen bond with Ser-125 having a bond length of 2.0 Å. Furthermore, one oxygen atom of the 1,3-dimethoxybenzene ring acted as an acceptor involved in hydrogen bonding with Asp-74 having a bond length of 2.4 Å, and the second oxygen atom of the methoxy group formed another hydrogen bond with aromatic amino acid Tyr-124 with a bond length of 2.0 Å of the peripheral anionic binding site (Figures 3A,B). These results were comparable with those of galanthamine (IC_50_ = 26.03 ± 0.4 µM, docking score = −17.301) which was used as a standard inhibitor of AChE. Gly121, Gly 122 of the oxyanion subsite and Tyr-124 of the peripheral anionic site (PAS) were found to be involved in the stabilization of the galanthamine-AChE complex. Similarly, Tyr-124 is involved in hydrogen bonding with the methoxy group of galanthamine with a bond length of 2.8 Å. Furthermore, Gly-121 and Gly-122 formed two hydrogen bonds with the same hydroxyl group of galanthamine with bond lengths of 1.8 and 1.9 Å, respectively (Figures 4A,B). The determined galanthamine inhibitory potential was found to be higher than compound **11**, as it is previously reported that, it may be due to the fact that galanthamine has interactions with all the four subsites (anionic and esteratic subsites) of the active site of the AChE (Choudhary et al., 2005). Whereas compound **11** has interactions mainly with PAS and does not interact with the amino acid residues of the catalytic traid (His-447, Ser-203, Glu-334). Therefore, compound **11** displayed a non-competitive type of AChE inhibition. The kinetic measurements agreed with the docking results, indicating compound **11** as a non-competitive inhibitor of AChE.

**FIGURE 5:**
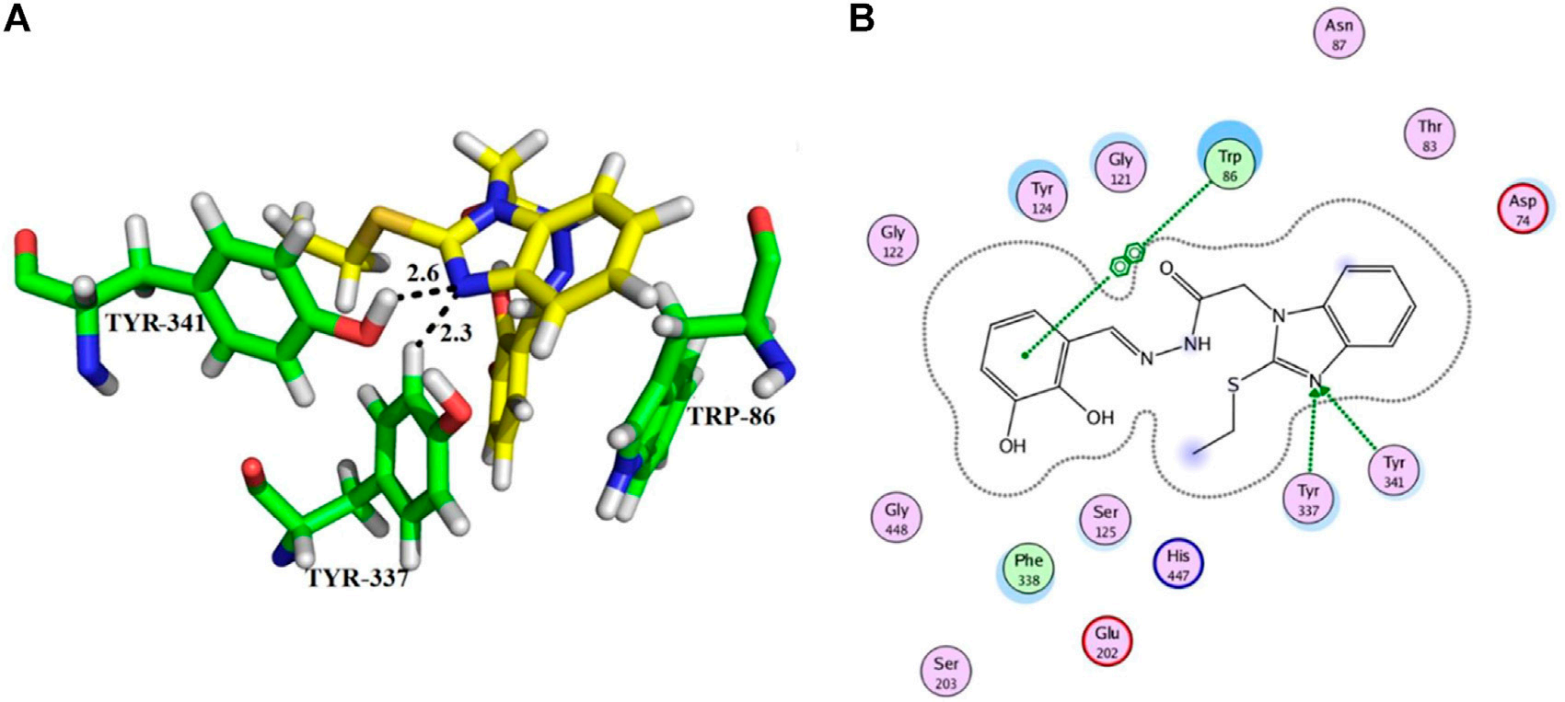
Showing the molecular docking conformation of compound **13** (**A,B**) in the active site of AChE.

From the docking analysis of other members of the series, compound **13** was the second most active compound with a docking score −14.560 and an IC_50_ = 50.58 ± 0.3 µM. This compound also displayed good inhibitory activity but was less potent than the standard galanthamine (Table 1). The compound **13** formed two hydrogen bonds and one arene-arene interaction with Tyr-337, Tyr-341, and Trp-86, respectively. In the case of compound **13**, the pyrocatechol ring formed π-π stacking interaction with Trp-86 of the anionic subsite. While Tyr-337 of the anionic site and Tyr-341 of PAS subsite established strong hydrogen bonds with the imidazole ring of the same compound having bond lengths 2.3 and 2.6 Å, respectively, as shown in Figures 5A,B. Compound **13** is slightly less potent as compared to compound **11**; it may be due to one less hydrogen bonding in this compound. All the interactions of compound **13** are with anionic subsites; therefore, docking results agree with the experimental kinetic data, indicating a non-competitive type of inhibition.

**FIGURE 6:**
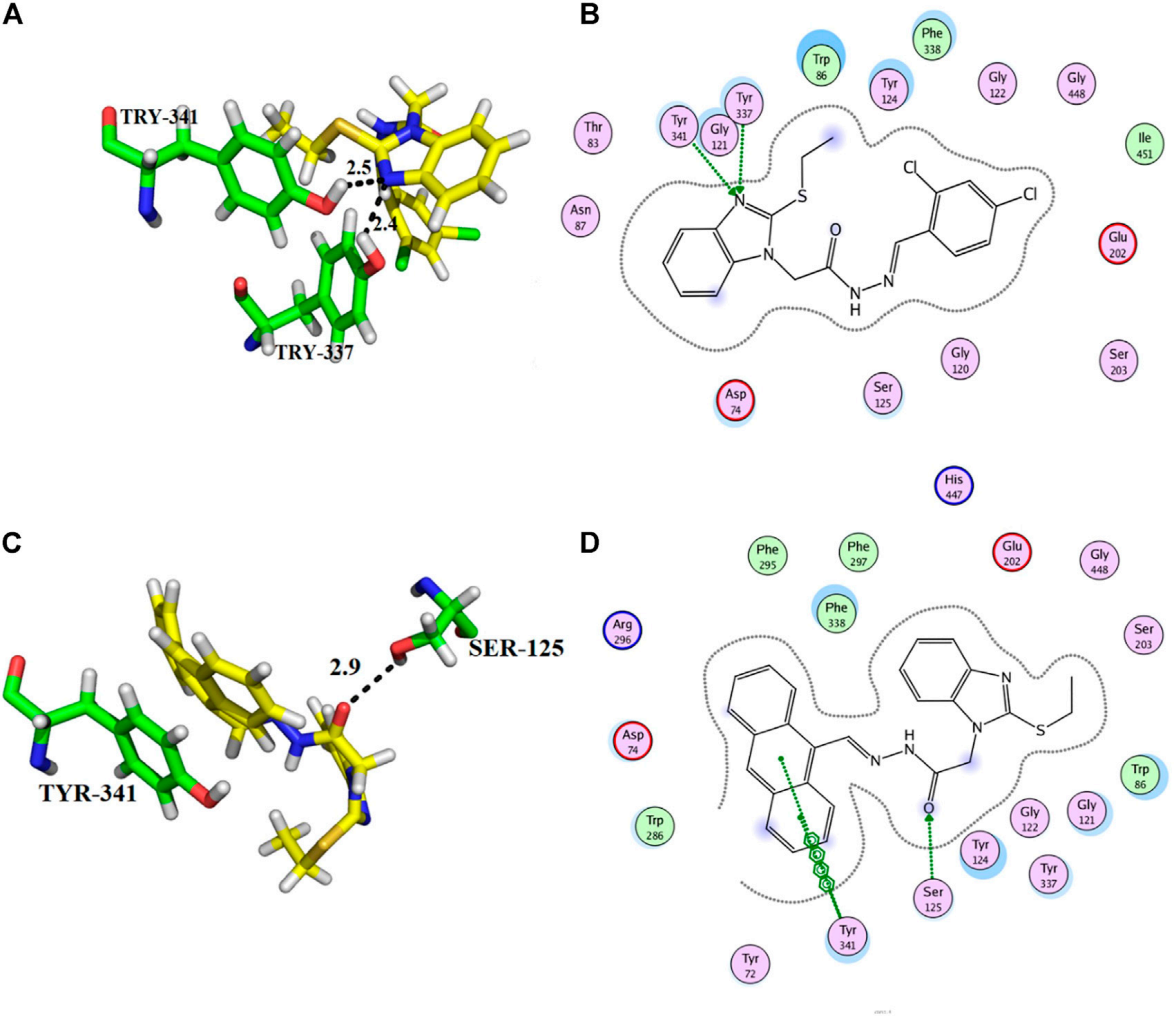
Showing the docking conformation of compound **10 (A,B)** and **14 (C,D)** in the active site of AChE.

Compounds **10** and **14** were the third most active members of the series, with the same IC_50_ = 51.23 ± 0.2 µM) and docking scores −12.950 and −12.572, respectively. These compounds also showed good inhibitory potential but were less potent as compared to standard galanthamine against AChE. From docking studies, it was noted that compound **10**’s imidazole ring formed two strong hydrogen bonds with Tyr-337 and Tyr-341, having bond lengths of 2.4 and 2.5 Å, respectively (Figures 6A,B). Compound **14** also mediated two polar interactions π-π interactions and hydrogen bonding with Tyr-341 and Ser-125 of the binding pocket. Ser-125 formed a strong H-bond with the carbonyl oxygen of the same compound, having a bond length = 2.9 Å. Tyr-341 also established two π-π linkages with anthracene moiety of compound **14**, as shown in Figures 6C,D. As all the four subsites are not involved in interactions with the ligand, therefore, docking results and kinetic experiments agree with each other, indicating both compounds are non-competitive inhibitors of AChE.

### 3.4 MD simulations

Molecular docking gives the representation of the static conformation of the protein-ligand complex (Hassan et al., 2022). However, the ligand continuously moves in the pocket of the enzyme. Therefore, to observe the motion of ligands in the enzyme’s active site, we performed MD simulations to explore different conformations and check the stability of our inhibitor-AChE complexes. Out of the six compounds, two potent compounds, **11** and **13,** were selected for MD simulation analysis.

**FIGURE 7:**
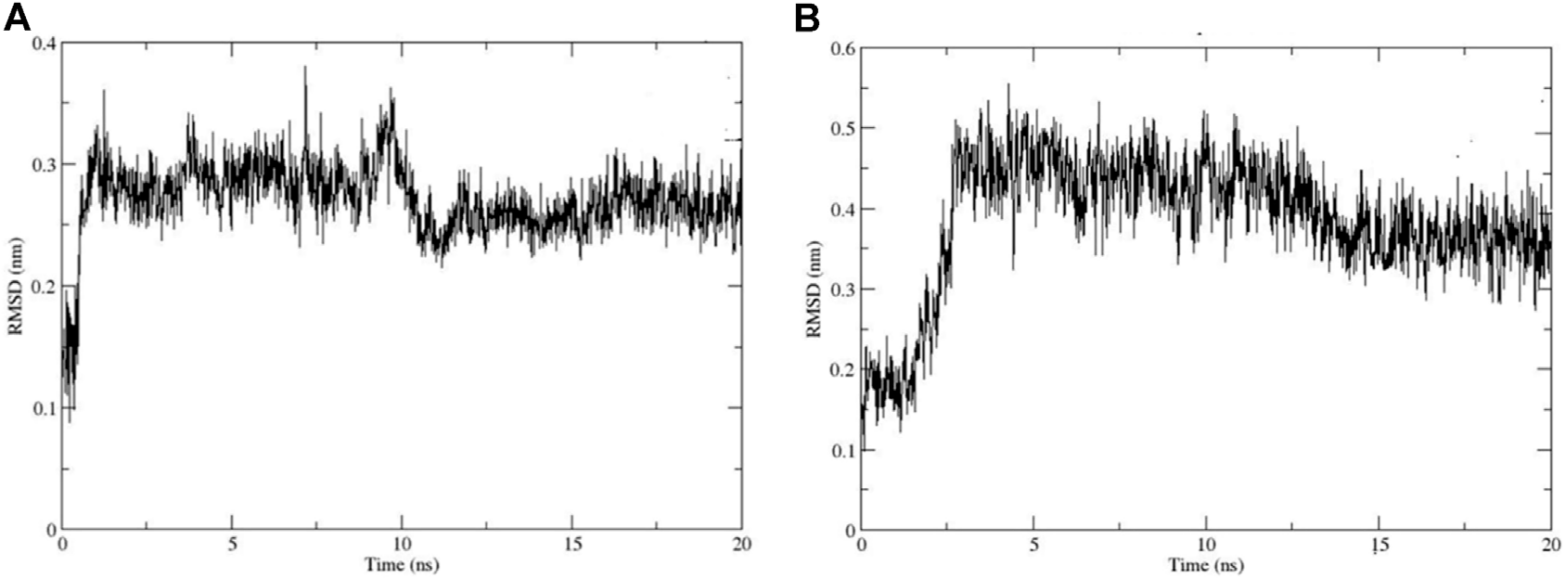
RMSD Plots for **(A)** Compound **11**-AChE complex **(B)** Compound **13**-AChE complex.

The stability of selected protein-ligand complexes was analyzed using RMSD, root mean square fluctuations (RMSF), and radius of gyration (Rg). The RMSD values of the complex backbone atoms were calculated to analyze the stability of the simulation process. Our results showed that the amplitude of the RMSD deviation curve for compound **11** in the complex with AChE is better in comparison to the stability of the compound 13-AChE complex. The smaller deviation curve indicates high stability and vice versa. Slight fluctuations were observed in the RMSD values for both the compound **11**-AChE and compound 13-AChE complexes during the initial 5 ns. However, after 10 ns, both the complexes began to stabilize, which indicates the stable behavior of the compounds by strongly inhibiting the target and showing explicit binding to the active site (Figures 7A,B).

**FIGURE 8:**
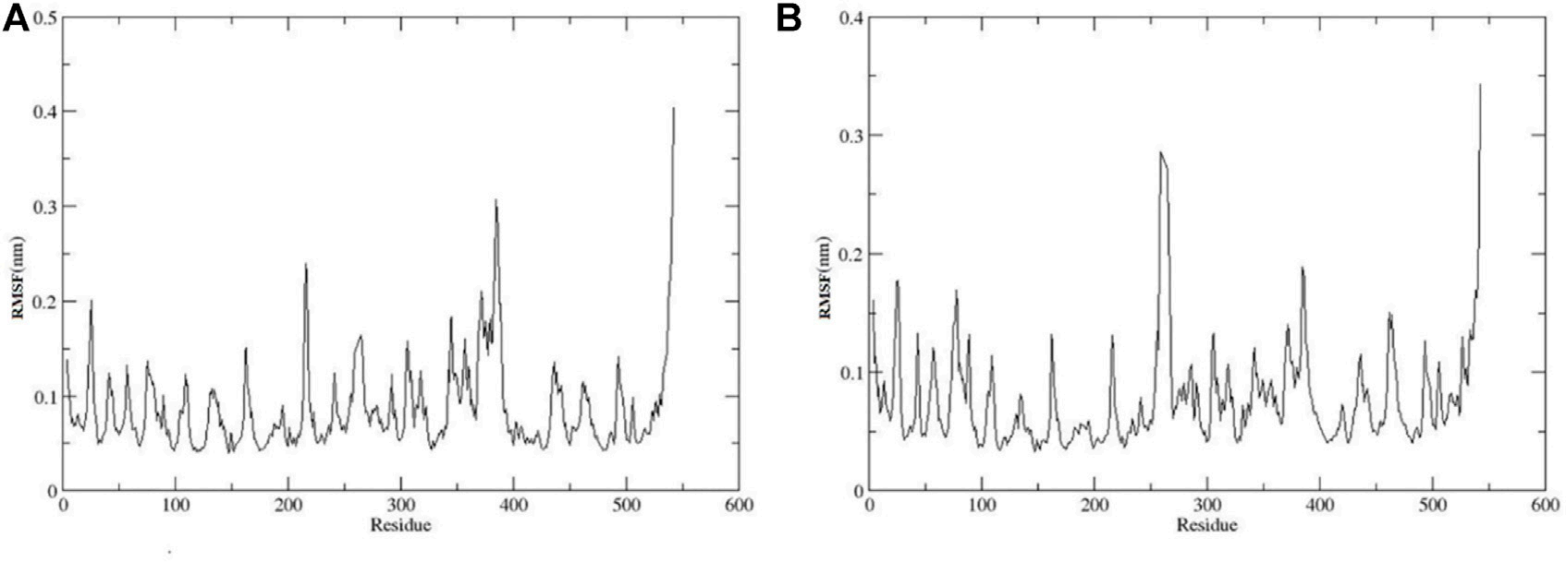
RMSF Plots for **(A)** Compound **11**-AChE complex **(B)** Compound **13**-AChE complex.

Afterward, we analyzed the RMSF to investigate the fluctuations of each residue of our enzyme in complex with both compounds **11** and **13** during simulations. For both the complexes, higher fluctuations were observed in the highly pliable regions of the AChE protein, including the –N and –C terminal and loop regions (Gly 256, Cys 25, Arg 274, Thr 275, Arg 276, Pro 277, Asn 283, His 284, Glu 285, Trp 286, and His 287) (Figures 8A,B).

**FIGURE 9:**
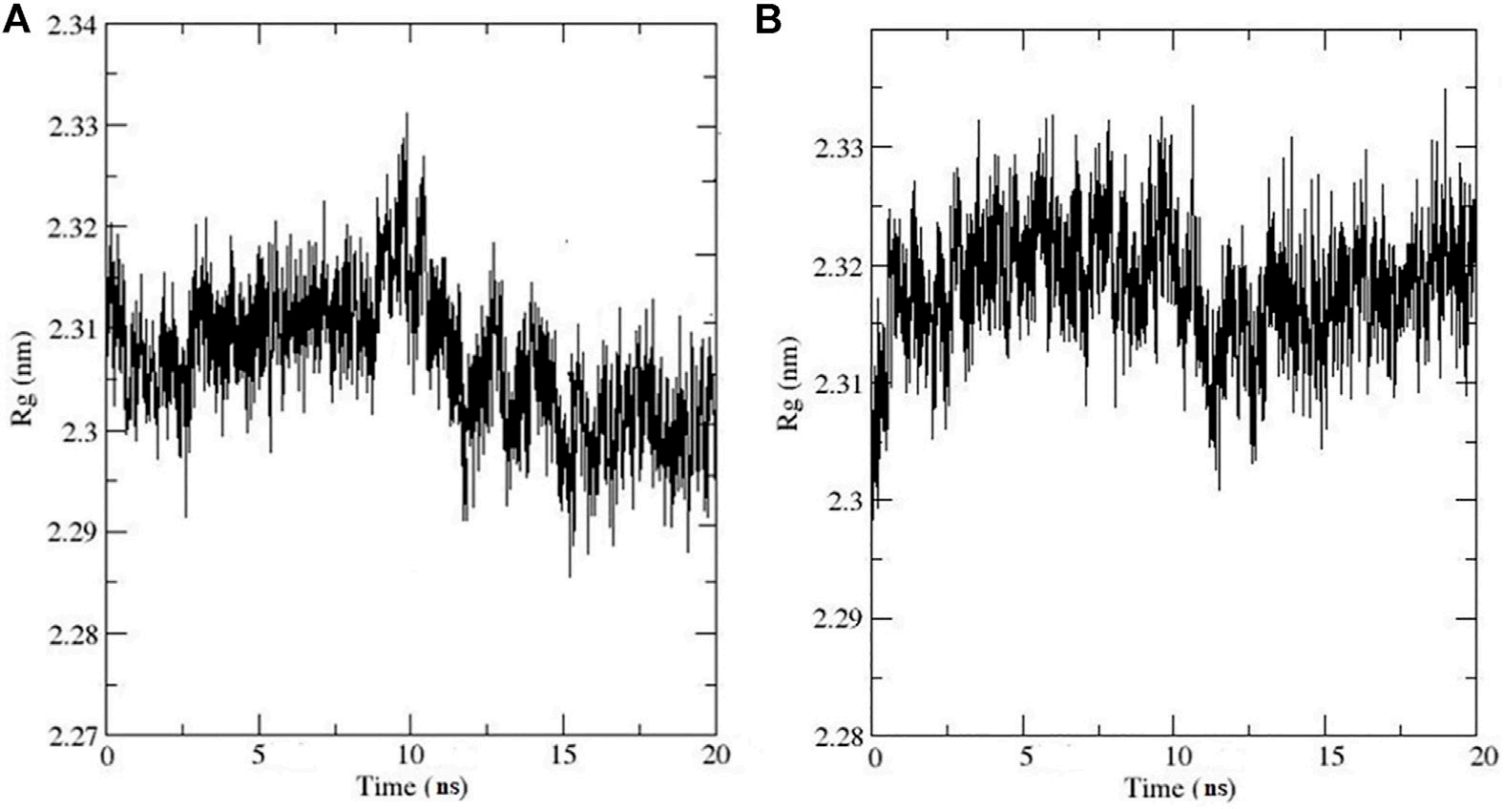
Rg Plots for **(A)** Compound **11**-AChE complex **(B)** Compound **13**-AChE complex.

In addition, the Rg was used to measure the structural compactness of ligands and interactions with the tertiary structural volume and is also utilized to determine the stability of the protein in the biological system along the MD trajectories. For the selected complexes, our result shows that the Rg values did not fluctuate considerably, as shown in Figures 9A,B, which indicates that the complexes remained compact upon ligand binding.

**FIGURE 10:**
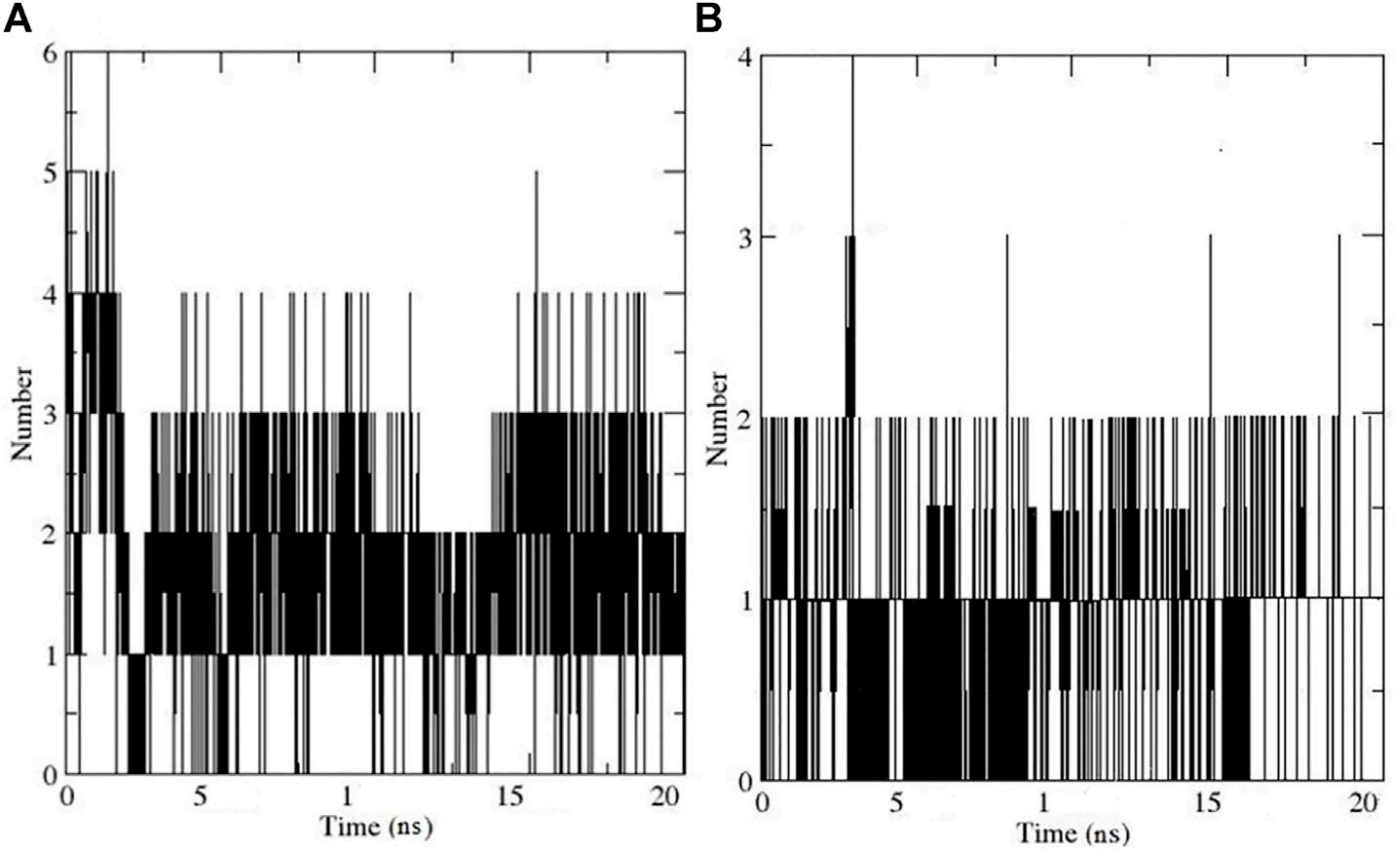
Illustrates the Hydrogen bonding for **(A)** Compound **11**-AChE complex **(B)** Compound **11**-AChE complex.

The hydrogen bond (HB) analysis was performed to assess the stability of the selected compound-AChE complexes. In general, both complexes retained HB interactions throughout MD simulation. In the case of the compound **11**-AChE complex, 0 to 6 HB were observed throughout the MD, while the compound **13**-AChE complex showed 0 to 4 HB. The current study result revealed that compound **11**-AChE complex possesses a maximum number of HB thus showing better stability in comparison to the compound **13**-AChE complex (Figures 10A,B). Overall, the stability analysis through RMSD, RMSF, Rg, and HB supports the high stability and inhibitory potential of compound **11** as compared to compound **13**.

### 3.5 Spasmolytic and calcium antagonistic activities

In the current study, all compounds of Schiff bases showed an antispasmodic effect by inhibiting the spontaneous contraction at different concentrations (Table 3). Among all the analogues, compound **11** was the most efficient, with an effective dose (ED_50_) value, 326.239 ± 0.01 µM (mean ± SEM, *n* = 3).

**TABLE 3:** Effective Dose (ED_50_) values of the compounds **9–14** for their spasmolytic effect on spontaneously and high K^+^ contracted isolated rabbit jejunum preparations.

Compounds	Spontaneous (µM)	High K^+^ (µM)
**9**	733.572 ± 0.01	2,257.145 ± 0.03
**10**	662.869 ± 0. 00	1,620.347 ± 0.04
**11**	326.239 ± 0.01	727.765 ± 0.09
**12**	702.670 ± 0.00	1,932.342 ± 0.06
**13**	458.926 ± 0.01	836.865 ± 0.05
**14**	592.876 ± 0.12	980.526 ± 0.05

Schiff bases were also evaluated at high K^+^ (80 mM) contracted isolated rabbit jejunum preparations for their spasmolytic effect, which was relaxed by all compounds (Table 3). Compound **11** again exhibited the highest potential with an ED_50_ value of 727.765 ± 0.09 µM (mean ± SEM; n = 3), indicating a Ca^+2^ antagonist effect. Schiff bases represent a class of compounds showing a wide range of bioactivities, such as antimicrobial and anticancer activities. Schiff base compounds are the products of primary amines and condensation of carbonyl compounds, which are extensively investigated owing to their wide range of biomedical applications (Sztanke et al., 2013). Amines have been found to exhibit antiproliferative activity against numerous cancer cell lines (Gama et al., 2011; Abu Bakr et al., 2016), while the presence of an azomethine bond is reported to be vital for bioactivities (Da Silva et al., 2011). The antispasmodic and Ca^+2^ antagonist activity of Schiff base compounds was first reported and could be linked to the azomethine bond.

### 3.6 Cytotoxicity

The cytotoxicity of 2-mercaptobenzimidazole hydrazone derivatives (**9–14**) on human neutrophils was determined. Galanthamine, a well-known inhibitor of AChE, was used as a standard drug. Results of human neutrophil viability (1 × 10^7^ cells/ml) against Schiff bases were shown in Table 4. Our results showed that all compounds have no toxic effects on human neutrophils like standard galantamine.

**TABLE 4:** Viability of human neutrophils (1 × 10^7^ cells/ml) in the presence of compounds **9–14**.

Compounds	Conc. µM	Viability [%]
**9**	564.286	50.54 ± 3.1
**10**	491.014	90.07 ± 2.3
**11**	501.907	98.02 ± 1.0
**12**	501.907	73.01 ± 3.0
**13**	539.913	77.22 ± 0.5
**14**	456.058	66.57 ± 4.0
Galanthamine	696.005	96.03 ± 2.0

## 4 Conclusion

In the current study, we evaluated the inhibitory potential and kinetic study of newly synthesized Schiff base class of 2-mercaptobenzimidazole hydrazone derivatives (**9–14**) and conformed their non-competitive inhibition. *In vitro* studies revealed the promising inhibitory potential of compound **11** against AChE. However, the potency of this compound was less as compared to standard galanthamine. These compounds also showed antispasmodic, Ca^2+^ antagonistic, and nontoxic effects on human neutrophils. These results suggested that Schiff base compounds could be used as a potential drug candidate against AChE to treat AD. Nevertheless, additional animal model-based studies are required to validate these results, which will help to design a new drug.

## Data Availability

The original contributions presented in the study are included in the article/Supplementary Materials, further inquiries can be directed to the corresponding authors.

## Abbreviations

Ach, acetylcholine; AChE, acetylcholinesterase; AD, Alzheimer’s disease; DTNB, 5-dithiobis 2-nitrobenzoic acid; ED_50_, effective dose; ELISA, enzyme-linked immune-sorbent assay; ES, enzyme substrate; hb, hydrogen bonding; I-TASSER, iterative threading assembly refinement; MD, molecular dynamics; mhz, Megahertz; MOE, molecular operating environment; NMR, nuclear magnetic resonance; PAS, peripheral anionic site; PDB, protein data bank; RMSD, root mean square deviation; RMSF, root mean square fluctuations; TLC, thin layer chromatography
