# Supplementary material for: 2-Mercaptobenzimidazole clubbed hydrazone for Alzheimer’s therapy: In vitro, kinetic, in silico, and in vivo potentials
Source: Front Pharmacol. 2022 Aug 9;13:946134. doi: 10.3389/fphar.2022.946134 (PMC9428891; doi:10.3389/fphar.2022.946134)
Supplement: Supplementary file 1 [file DataSheet1.docx]

**2-Mercaptobenzimidazole clubbed hydrazone for Alzheimer's therapy: *In-vitro*, Kinetic, *in-silico* and *in-vivo* potentials**

Farida Begum^1^, Najeeb Ur Rahman^2^, Ajmal Khan^2^*, Sajid Iqbal^3^, Rehan Zafer Paracha^4^, Jalal Uddin^5^, Ahmed Al-Harrasi^2^*, Muhammad Arif Lodhi^1^*

^1^Department of Biochemistry, Abdul Wali Khan University Mardan, Khyber Pakhtunkhwa 23200, Pakistan [faridaaziz90@yahoo.com](mailto:faridaaziz90@yahoo.com) (FB) [arifbiochem@hotmail.com](about:blank) (M.A.L.)

^2^Natural and Medical Sciences Research Centre, University of Nizwa, Birkat-ul-Mouz 616, Nizwa, Sultanate of Oman [najeeb@unizwa.edu.om](mailto:najeeb@unizwa.edu.om) (NUR) [ajmalchemist@yahoo.com](mailto:ajmalchemist@yahoo.com) (AK) [aharrasi@unizwa.edu.om](mailto:aharrasi@unizwa.edu.om) (A.A-H.)

^3^Atta-ur-Rahman School of Applied Biosciences (ASAB), National University of Sciences and Technology (NUST) Islamabad 44000, Pakistan [sajidiqbalmb44@yahoo.com](mailto:sajidiqbalmb44@yahoo.com) (S.I.)

^4^Research Centre for Modelling & Simulation (RCMS), National University of Sciences & Technology (NUST), Islamabad 44000, Pakistan [rehanzfr@gmail.com](mailto:rehanzfr@gmail.com) (R.Z.P.)

^5^Department of Pharmaceutical Chemistry, College of Pharmacy, King Khalid University, Abha 62529, Saudi Arabia [jalaluddinamin@gmail.com](mailto:jalaluddinamin@gmail.com) (JU)

**Chemistry**

The synthesis of 2-Mercaptobenzimidazole derivatives (**9-14**) were carried out through multistep reactions. First, 2-Mercaptobenzimidazole was refluxed with bromoethane using necessary condition (KOH) in ethanol with equimolar amounts for about 10 h. After completion of the reaction, the reaction mixture was filtered. The filtrate so obtained was kept until the whole ethanol was evaporated and got shiny white needle-like crystals of 2-ethylthio benzimidazole. In the second step 2-ethylthio benzimidazole was taken in round bottom flask refluxed with ethyl chloroacetate (dropwise) using anhydrous potassium carbonate in DMF (solvent) for about 15 h. After completion of the reaction, semisolid product (2-(2-(ethylthio)benzimidazolyl) acetate) was obtained. In the third step, 2-(2-(ethylthio)benzimidazolyl) acetate was refluxed in methanol with hydrazine hydrate for about 10h. The product, 2-((ethylthio)benzimidazolyl) acetohyrazide get was poured into cold water until a precipitate was formed. The precipitate was filtered and then dried in an open atmosphere. In step, the fourth 2-((ethylthio)benzimidazolyl) acetohyrazide was dissolved in methanol with 2-3 drops of glacial acetic acid (catalyst) on the hotplate. After 10 minutes, aldehyde was added and refluxed to the whole mixture for about 5-6 h. TLC monitored the progress of the reaction. After completion of the reaction, the mixture was poured into cold water until a precipitate was formed. The precipitate was collected by filtration, washed with water and then dried in an open atmosphere.

**Scheme-1:** Synthesis of 2-Mercaptobenzimidazole hydrazone derivatives **9-14**


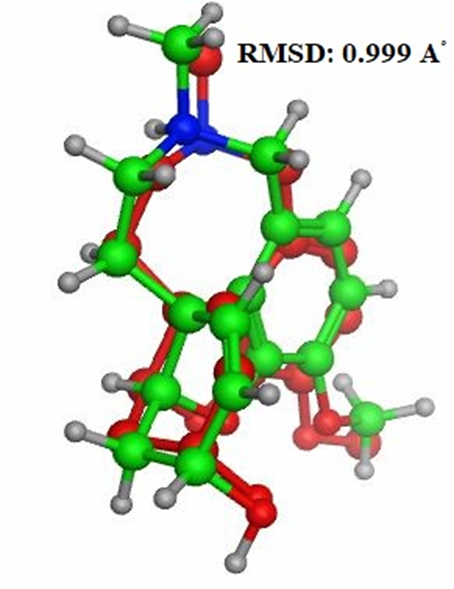


**Figure S1**. Superposition of co-crystalline ligand conformation (Red) and docked conformation (Green)
